# PA and PA-X: two key proteins from segment 3 of the influenza viruses

**DOI:** 10.3389/fcimb.2025.1560250

**Published:** 2025-03-14

**Authors:** Xin Zhang, Yingying Tao, Li Wu, Jianhong Shu, Yulong He, Huapeng Feng

**Affiliations:** ^1^ Department of Biopharmacy, College of Life Sciences and Medicine, Zhejiang Sci-Tech University, Hangzhou, China; ^2^ Zhejiang Provincial Engineering Research Center of New Technologies and Applications for Targeted Therapy of Major Diseases, College of Life Sciences and Medicine, Zhejiang Sci-Tech University, Hangzhou, China; ^3^ Department of Biology, College of Life Sciences, China Jiliang University, Hangzhou, China

**Keywords:** influenza viruses, PA, PA-X, host factors, antiviral drugs, host shutoff activity, innate immunity

## Abstract

In recent years, the influenza viruses have posed an increasingly severe threat to public health. It is essential to analyze the virulence and pathogenesis of influenza viruses to prevent and control them, as well as create antiviral drugs. Previous studies have revealed that influenza virus segment 3 codes for not only the PA protein but also a novel protein, PA-X. PA protein is one subunit of the polymerase of influenza viruses and plays a critical role in its life cycle. PA presented endonuclease activity, the transcription and replication of the viral genome, viral virulence, protein degradation, and host immune response by interacting with viral proteins, including PB2, PB1, and host factors, including ANP32A, CHD6, HAX1, hCLE, HDAC6, MCM complex. PA mutations were involved in the viral replication, pathogenicity, and transmission of influenza viruses in poultry, mammals, and humans. PA-X is an open reading frame generated by +1 ribosomal code shift at the N-terminal amino acids of segment 3 and possesses the shutoff activity of host gene expression, regulating the host immune response, viral virulence and transmission. Therefore, PA is one ideal target for the development of antiviral drugs against influenza viruses. Baloxavir marboxil (BXM) and Favipiravir are two very effective anti-influenza virus drugs targeting the PA endonuclease domain of influenza A viruses. In this review, we summarized the structures, viral replication, virulent determinants and transmission, host factors, innate immunity, and antiviral drugs involved in PA and PA-X. The information is of great value for underlying the mechanism of viral replication and developing novel effective strategies to prevent and control influenza infection and the pandemic.

## 1 Introduction

Influenza virus belongs to the family Orthomyxoviridae and is an enveloped, segmented, single-stranded RNA virus (Braam et al., 1983; Brown et al., 2001). It can cause regular annual epidemics and re-emerging pandemics, posing a potentially serious threat to public health and the global economy (Chu et al., 1949). Influenza viruses have a wide range of hosts and can infect wild birds, poultry, and some mammals such as dogs, horses, and humans (Imai et al., 2012; Zhang et al., 2013). The human influenza virus usually causes coughing, fever, muscle aches, and limb weakness. Certain subtype strains (e.g., H5N1, H7N9) are more pathogenic and cause more severe clinical symptoms, even death (Imai et al., 2012; Watanabe et al., 2013). Meanwhile, avian influenza viruses are highly contagious and can spread rapidly among poultry flocks, resulting in the death of large numbers of birds or even the extinction of entire flocks (Yuen et al., 1998). In 2024, the H5N1 avian influenza virus was detected for the first time in the United States in cows (Gu et al., 2024; Lin et al., 2024). This H5N1 virus quickly spread to dairy herds in 11 states, and bovine-to-human transmission subsequently occurred (Garg et al., 2024). As of 17 January 2025, there have been 67 cases of human infection with H5N1 avian influenza viruses from dairy cattle (Song et al., 2025). The US Centers for Disease Control and Prevention (CDC)also found that the viral hemagglutinin (HA) protein from California’s recently confirmed H5N1 avian influenza cases had undergone different changes, and HA glutamine(Q)226leucine(L) mutation enhanced virus replication (Lin et al., 2024). Influenza viruses can be categorized into influenza A viruses, influenza B viruses, influenza C viruses, and influenza D viruses according to the antigenic proteins on their surface, of which influenza A viruses are the most common and the most widely transmitted viruses among humans and animals (Dimmock et al., 1980; Su et al., 2017). A new influenza virus, called influenza virus D(IDV), was isolated from swine in 2011 and subsequently spread in Bovine. Evolutionary analysis of the complete genome showed that IDV has two circulating lineages that can undergo recombination, and significant antigenic differences were observed between the two lineages (Hause et al., 2013; Collin et al., 2015). The influenza virus genome consists of eight single-stranded RNA segments, each encoding one or more proteins, and the length of the eight RNA genomes is approximately 13,500 nucleotides (Chen et al., 2001; Renzette et al., 2014). IAV can be divided into 18 HA and 11 NA subtypes based on HA and neuraminidase (NA) proteins (Chaiyawong et al., 2022). Polymerase basic protein 2(PB2), Polymerase basic protein 1(PB1), and Polymerase acid protein (PA) subunits constitute the influenza virus polymerase complex, which is responsible for the viral transcription and replication (Pflug et al., 2014). HA is a glycoprotein on the surface of viruses and is divided into HA1 and HA2. HA1 is mainly responsible for binding to sialic acid receptors on the surface of host cells, mediating viral attachment and entry. HA2 is primarily involved in the fusion of viruses with the cell membranes of the host cells and assists in the entry of viral RNA into the host cells (Yamada et al., 2006). Nucleoprotein (NP) is an important component of the influenza virus. As a structural protein within the influenza virus, it acts together with the influenza virus RNA polymerase (PB2, PB1, PA) to form a transcription complex that facilitates influenza virus transcription and replication (Turan et al., 2004). Furthermore, NP is also a triple-helical structure with a strong affinity for viral RNA, forming a ribonucleoprotein complex (RNP) (Hao et al., 2008). The NA of the influenza virus is also a crucial transmembrane glycoprotein on the surface of the influenza virus, and together with the HA protein, it constitutes the two major surface antigens of influenza viruses, and the enzyme active portion consists of four identical subunits, which are bound by non-covalent bonds to form a tetrameric structure. It facilitates virus release and transmission by hydrolyzing sialic acid on the host cell surface (Barman and Nayak, 2000). The inner layer of the envelope is composed of matrix protein 1 (M1) and matrix protein (M2), M2 is an ion channel protein (Ito et al., 1991). Non-structural protein 1 (NS1) is a key virulence factor that influences influenza pathogenesis and adaptation to new hosts (Basler et al., 2001). Influenza viruses are highly mutable, especially the influenza A virus (H3N2), which undergoes genetic recombination and mutation, causing a significant impact on human health (Lee et al., 2018).

Segment 3 mainly transcripts two mRNAs encoding PA and PA-X, which play crucial roles in the viral life cycle of influenza viruses (Huarte et al., 2003; Jagger et al., 2012). The PA-X protein, as a variant of the PA protein, has the same first 191 amino acids as PA, but its C-terminal structural domain (61aa) is completely different due to ribosome translocation (Shi et al., 2012). PA-X is implicated in suppressing host gene expression and the immune response, especially through the interferon-related gene pathway mRNAs and viral adaptation (Hayashi et al., 2015). In this review, we summarized the structure and function of PA and PA-X, innate immune regulation, interacting host factors, and antiviral drugs in order to provide valuable information for the prevention and control of influenza viruses.

## 2 Structure and composition of PA and PA-X

**Figure 1:**
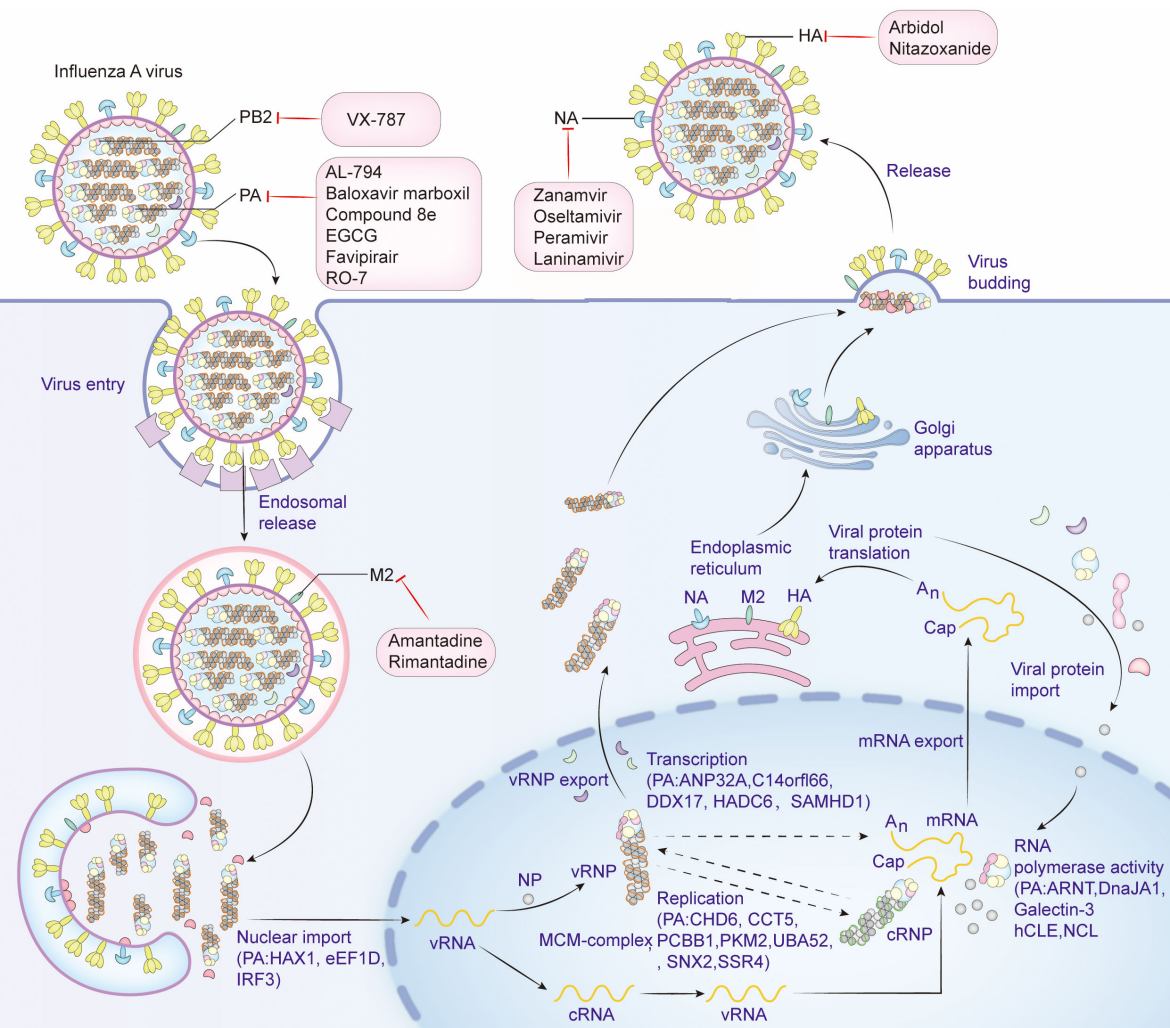
Life cycle of influenza virus, host factors that interact with PA, and targets of antiviral drugs. Viral attachment (Attachment): The influenza virus binds to the host cell surface via the HA protein on its surface, and the initial attachment of the virus to the host cell is completed by binding to the sialic acid receptor on the surface of the host cell. Endocytosis: Once the virus binds to the receptor on the host cell surface, the cell membrane envelops the virus-receptor complex to form an endocytic vesicle and enter the host cell. Acidification and Fusion: The acidic environment inside the endocytic vesicle causes a conformational change in the HA protein, and the virus fuses with the endocytic vesicle membrane, releasing the viral genome into the cytoplasm. RNA Release and Transcription: The influenza virus RNA genome is released into the cytoplasm and then enters the nucleus, where transcription and replication occur to produce mRNA and new viral genome RNA. Protein Synthesis: In the cytoplasm, viral mRNA is translated into viral proteins, including newly synthesized HA, NA and viral core proteins, by the host cell’s ribosomes. Viral Assembly: The newly synthesized viral genome from replication and transcription and the synthesized viral proteins HA, NA and M2 are modified by the endoplasmic reticulum and the Golgi apparatus in the cytoplasm, and then assembled with other viral proteins to form new virus particles. Virus budding (Budding): The newly formed virus particles are released into the extracellular space through a budding process from the host cell membrane. The NA is used to cleave the sialic acid residues on the surface of the host cell, helping the virus release and infect new cells. Antiviral drugs that inhibit influenza virus proteins and the host factors that interact with PA are shown in detail in the figure. Create with Adobe Illustrator 2023.

PA protein is the main product encoded by segment 3 (Parvin et al., 1989). It comprises 716 amino acids and weighs about 85.5 kDa in molecular weight. PA proteins is located inside the viral particles and has a negative charge (Hara et al., 2006; Pflug et al., 2014). PA can be cleaved into two parts: PA-N (1-256) and PA-C (277-716), connected by a “linker” from position 257 to 276 (He et al., 2008; Liu et al., 2009). The influenza virus polymerase is composed of the PA, PB1, and PB2 subunits, it also forms viral ribonucleoprotein complex (vRNP) together with the NP protein and plays an important role in the assembly of viral particles. The PA protein mainly resides in the nucleus of the host’s infected cell (Biswas et al., 1998). The N-terminal domain of the PA protein has the capability of an endonuclease and can restrict the production of host protein and induce apoptosis in virus-infected cells, thus being an apt target for anti-influenza drug development (Dias et al., 2009; Datta et al., 2013). Further, Nogales et al. reported that the functional region responsible for the inhibition of host protein synthesis was mapped to the region spanning amino acid residues 57 to 114 close to the easily changeable ring structure of PA protein and a region of α spirals (Nogales et al., 2017). The PA protein of A/California/04/2009 (H1N1) (CA04) possesses a more potent ability in host protein expression inhibition than that of A/WSN/33 (H1N1, WSN) (Gao et al., 2014). The structural domain within the N-terminus of the PA protein interacts with PB2 subunits (Datta et al., 2013). This region is important for the assembly of the polymerase complex. The C-terminal region of PA directly interacts with the N-terminus of PB1, which is important for the stability and function maintenance of the polymerase complex (Dias et al., 2009). The interaction domains of the PA protein are confined within amino acids 239-716, and those of the PB1 protein encompass amino acids 1-15. The region consisting of amino acid Q at position 670 of PA protein and amino acids phenylalanine(F) at position 9, valine(V) at position 12, and proline(P) at position 13 of PB1 protein could be a potential interaction site (Zürcher et al., 1996; Mizuta et al., 2022). The PA subunit T515 site is located at its C-terminal end, at the binding site of the PA-PB1 interaction (Liu et al., 2009). In addition, the serine(S)421, arginine(R)443, lysine(K)497, histidine(H)510, K539, threonine(T)552, R638, S659, and K665 key sites of PA are also located in the region where PA interacts with PB1 (Zhao et al., 2016). However, there is no direct interaction between PA and PB2; the interaction between PA and PB2 is indirect, and they work together in the RNA-dependent RNA polymerase complex of influenza viruses through the bridging action of PB1. Although there is no direct strong binding between them, their interaction is important for RNA synthesis and viral replication (González et al., 1996; Perez and Donis, 2001; Deng et al., 2005). The first 100 amino acids of the PA subunit interact with PB2, e.g., S37, V63, and T97 (Hemerka et al., 2009). The N-terminal structure of the PA protein has an α/β architecture with five mixed β-strands (β1-5) forming a twisted plane surrounded by seven α-helices (α1-7). A strongly negatively charged cavity formed by multiple acidic residues is surrounded by helices α2-α5 and chain β3 and contains a metal binding site (He et al., 2008). Each step of the influenza virus life cycle is essential. Host factors interacting with PA and antiviral drugs can be mapped at each step. (**Figure 1**).

In 2012, Jagger et al. discovered a highly conserved sequence (UCCUUUCGUC) in the gene segment 3 of the influenza A virus, potentially linked to the ribosomal open reading frame’s movement. Moreover, gene segment 3 encodes the PA protein and also encodes a new type of protein, PA-X, which was previously unknown (Jagger et al., 2012). The synthesis of PA-X protein firstly uses the initial codon of PA protein to start translation, synthesizes the N-terminal including 191 amino acids of PA protein, and then recognizes X-ORF encoding a peptide segment of 61 amino acids through +1 ribosomal frameshifting (Desmet et al., 2013). The N-terminal PA-X protein, responsible for breaking down intracellular mRNA templates, has a much more powerful effect on diminishing host cell protein expression than its N-terminal equivalent (Hayashi et al., 2015). A transformation in the translation reading frame may be the cause of the C-terminal of the PA-X protein being distinct from that of PA, potentially allowing the influenza virus to elude the host antiviral response. It is believed that ribosome code shifting during the translation of PA mRNA is the source of its production (Shi et al., 2012). In the process of translation, the ribosome moves on the PA mRNA. When it passes through a region rich in U, it is followed by a rare codon (Firth et al., 2012). Because the decoding is slow, the rare codon promotes the ribosome to +1 code shifting, which makes the codon CGUCAG interpret as GUC, leading to the shift of the open reading frame, which is called X-ORF (Jagger et al., 2012). PA-X protein exists in two forms: full-length and truncated. In most cases, it is expressed in its full-length form. It consists of the N-terminal 191 aa of the PA protein and the C-terminal 61 aa encoded by the X-ORF. However, in some cases, PA-X is expressed in a truncated form, consisting of the N-terminal 191 aa of the PA protein and the C-terminal 41 aa encoded by the X-ORF (Shi et al., 2012; Hussain et al., 2019). Mutational analysis showed that proline and serine at positions 28 and 65 play a central role in this difference, and P28 and S65 contribute to the enhanced shutoff activity of PA-X (Oishi et al., 2019). PA-X 100V significantly increases viral polymerase activity and viral replication in mammalian cells. At the same time, PA-X 100V significantly enhances the adaptability of the virus in mice and reduces its toxicity in chickens (Hussain et al., 2019; Chen et al., 2025). The R195K, K206R, and P210L substitutions in the PA-X protein significantly enhance the pathogenicity and transmissibility of the H9N2 virus in mice and ferrets (Sun et al., 2020; Chiem et al., 2021). The above are the main active sites of PA-X that were recently discovered, which laid the foundation for a deeper understanding of PA-X.

## 3 Functional properties and immunity of PA and PA-X protein

**Figure 2:**
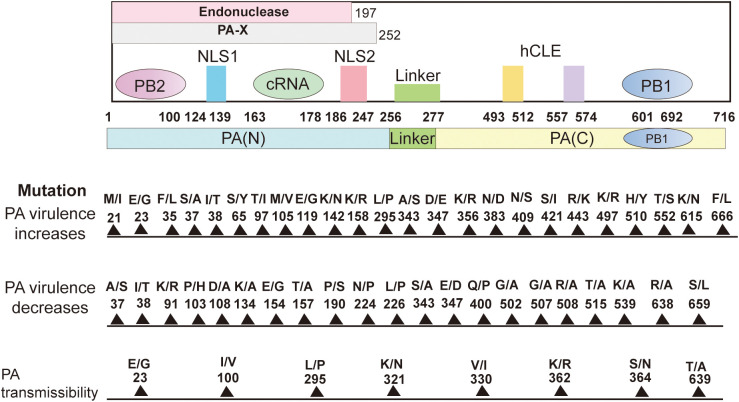
Structure of the PA subunit and host-adaptive mutation sites. It comprises 716 amino acids. PA can be cleaved into PA-N (1-256) and PA-C (277-716) and connected by a “linker(257-276). The amino acids 1-197 of PA-N have endonuclease activity. The nuclear localization signal is located in the 124-139 and 186-247 amino acids (aa) peptide of PA-N. The first 100 amino acids of PA-N can interact with PB2. The amino acids 668-692 of the PA-C amino acid(aa) motif bind to PB1-N 1-12 amino acids. The PA-X N-terminal 191 amino acids are repeated with PA, and the full-length PA-X contains a sequence of 1-252 amino acids. 493-512 and 557-574 of PA protein can interact with host protein hCLE. Site-specific mutations in the PA protein affect polymerase activity, replication, pathogenicity, transmission, and host inflammatory response and can enhance or reduce viral virulence. Create with Adobe Illustrator 2023.

PA plays several key roles in the life cycle of influenza viruses, especially in the replication, transcription, and regulation of the viral genome (Kawaguchi et al., 2005). The PA protein is the catalytic core of the RNA-dependent RNA polymerase complex (RdRp) of influenza viruses and is responsible for the synthesis of viral RNA (Liu et al., 2009). The PA protein, together with PB1 and PB2, constitutes the RNA polymerase of influenza viruses and is involved in two key processes: replication of genomic RNAs and mRNA synthesis (Kawaguchi et al., 2005). PA interacts with PB1 to maintain RNA stability (Perez and Donis, 2001). The binding of PA to PB2 is not as strong as that of PB1, but PA is involved in RNA synthesis through the collaboration of PB1 and PB2. Influenza virus initiates transcription by the cap-snatching mechanism, the viral RdRp interacts with the C-terminal domain (CTD) of host RNA polymerase II (RNAPII) (Tarendeau et al., 2007). PB2 is mainly responsible for binding to the host cell’s mRNA 5’ cap structure, the captured host cell mRNA is then cleaved by PA-N terminal for viral mRNA transcription. PA-Nter has DNA and RNA nucleic acid endonuclease activities that can be preferentially activated by manganese ions (Dias et al., 2009; Crépin et al., 2010). The endonuclease activity of 1-197 amino acids of PA-N can cleave 10-13 nucleotides on the 5’cap of the host pre-mRNA to obtain short RNA sequences as primers for viral mRNA transcription (Yuan et al., 2009). This is a key step in the cap-snatching process and is important for the translation and stability of viral mRNA. Subsequently, the PB1 subunit utilizes this primer to initiate viral mRNA synthesis and directs the transcription of viral mRNA by cap-snatching post-host RNA segment (Biswas et al., 1998). As mentioned previously, PA can be hydrolyzed by proteases into the N- and C-terminal, and PA-N contains amino acids 1-256, which is the main functional region of PA. PA-N has a nuclear localization function; PA proteins that are transcribed or translated into the cytoplasm need to be transferred to the nucleus (Nieto et al., 1994; Guo et al., 2023a). Amino acids 124-139 and 186-247 of PA-N are considered to be the nuclear localization signals of PA proteins, which can guide the PA proteins from the cytoplasm into the nucleus (Nieto et al., 1994). In addition, PA-N has protease activity that induces protein hydrolysis and is capable of degrading both host and viral proteins. PA-N recognizes and binds to the promoters of vRNAs and cRNAs, initiating transcription and replication of the viral genome (Sanz-Ezquerro et al., 1995). A deeper understanding of the structure of PA and PA-X and specific amino acid site mutations can help provide a better understanding of the influenza virus(**Figure 2**).

Amino acid substitutions in PA protein affect the replication of influenza viruses *in vitro*. PA K356R mutation enhanced the nuclear import efficiency, polymerase activity, and genome transcription and replication of the H9N2 virus in mammalian cells but not replication in avian-derived cells, suggesting that PA K356R is a mammalian cell-specific regulatory factor (Xu et al., 2016). PA S37alanine(A) mutation in H7N9 increases polymerase activity and enhances viral replication in human A549 cells (Yamayoshi et al., 2014). PA F666L substitution enhances H7N7 influenza virus replication in MDCK cells, and the PA F666 site is also located in the PA-PB1 interaction region, indicating that PA F666L may enhance the binding of PA and PB1 (de Wit et al., 2010). The PA T97isoleucine(I) and PA I545V variants antagonize live virus replication in A549 cells, and the antagonistic effect of PA T97I and PA I545V on recombinant virus replication is compensated when these proteins interact with PA S594glycine(G). The C-terminal structural domain of the PA protein is also the binding site of the host transcription factor hCLE and binds to PB1. PA I545V and PA S594G are proximal to the hCLE binding site (Wan et al., 2022). Thus, it is postulated that PA I545V and PA S594G might affect RNA synthesis modification by altering the interaction between PA and hCLE. Recent studies showed that T97I, I545V, and S594G mutations in PA result in enhanced polymerase activity in mammalian cells (Ma et al., 2024). P103H and S659L PA mutations in H7N7 resulted in significantly reduced replication in A549 and MDCK cells. The replication ability was 10 times lower than its counterpart (Zhao et al., 2016). The mutation of PA glutamic acid(E)206K reduced the ability for A/WSN/33 influenza viruses to replicate in mammalian cells and was considered a temperature-sensitive mutant. The mutation E206K could not translocate complexes of PA-PB1 into the nucleus at temperatures of 39.5°C and could only be virally active at the temperatures of 33°C and 37°C, but viral titer was undetected (Cao et al., 2024). On the other hand, The K362R and S364asparagine(N) mutations in PA reduced nuclear translocation efficiency and PA protein expression levels in the cytoplasm and nucleus of CEF cells (Xu et al., 2023). PA S224P and N383aspartic acid(D) enhance virulence in duck-origin cells, PA is the causative agent of H5N1 avian influenza (Song et al., 2011).

PA mutants play an important role in the pathogenicity of influenza viruses. PA T515 has a critical role in the previously described PA-PB1 binding site. The T515A mutation in H5N1 eliminated pathogenicity in wild birds, but replication and transmission were not affected, and this mutation affects the early course of infection of the virus. However, the single T515A mutation in PA did not affect the pathogenicity of the influenza virus in ferrets or mice (Hulse-Post et al., 2007). Zhong et al. characterized H5N1 viruses (QT1728, QT1480) isolated in Vietnam and revealed both are highly virulent in mice but significant differences in polymerase activity. The PA S343A/E347D mutation of the QT1728 virus reduced the viral polymerase activity and mouse pathogenicity. In contrast, the PA 343S/347E mutation increased the polymerase activity and virulence of QT1480, a low pathogenicity H5N1 influenza virus (Zhong et al., 2018). In an infected mouse model, PA K356R enhanced the expression of inflammatory factors by H9N2 AIV in the lungs, which showed significant inflammatory infiltration and pathologic damage (Xu et al., 2016). In addition to enhancing replication *in vitro*, PA S37A also significantly increased the pathogenicity of the mouse model *in vivo* (Yamayoshi et al., 2014). For example, the PA K615N mutation in H7N7 enhances mammalian polymerase activity and pathogenicity in mice, and N615 at a site of stronger PA-PB1 interaction (Gabriel et al., 2005, 2007). Other mutations, such as PA methionine(M)311I and PA A343S, enhance polymerase activity toward more viral production and higher levels of pro-inflammatory cytokines/chemokines, contributing to pathogenicity in mice (Guo et al., 2023b). P103H and S659L lower the pathogenicity of the virus in mice synergistically by reducing the activity of the polymerase. P103H is present in the hydrophobic region of PA-N. Similarly, the mutation PA S659L falls within the C-terminal region of PA at the interface with PB1. This makes it an ideal target for the development of attenuated vaccines and therapies against viruses (Zhao et al., 2016). PA E206K temperature-sensitive mutant reduces pathogenicity *in vivo* (Cao et al., 2024). Also, the replacement of the human PA subunit in the avian influenza virus polymerase can alleviate restrictions in human cells. Mutation analysis has shown that human PA protein activity enhancement is mainly due to the change of threonine to serine at residue 552. Compared with variants containing avian polymerase with human PA subunits, only the T552S mutation replicated faster in culture and was more pathogenic in mice than viruses containing only the avian polymerase complex (Mehle et al., 2012). PA S49tyrosine(Y), D347G, and PB2 S155N, these mutations result in a highly pathogenic virus and a mouse-adapted phenotype. Further studies showed that these three mutations increased polymerase activity, viral transcription, and replication in mammalian cells, severe interstitial pneumonitis, excessive inflammatory cell infiltration, and accelerated growth in mice (Guo et al., 2023a). The AIV PA T32M/L550I mutant has both reduced replication and virulence in infected mice and can be used as a target for the development of specific and effective anti-influenza therapies (Yang et al., 2023a).

PA mutations are involved in altering the transmissibility of influenza viruses. Ferrets are a golden model for evaluating the transmissibility of influenza viruses (Karlsson et al., 2015). Eurasian avian influenza-like H1N1 (EA H1N1) swine influenza viruses are widely circulating in pigs around the world, and LN265 and GZ828 are typical of the strains isolated from them. The four amino acids 100I, 321K, 330V, and 639T in PA are essential for the pathogenicity and efficient transmission of LN265 in ferrets. Replacement of these four amino acids in PA with 100V, 321N, 330I, and 639A decreases the ability of LN265 to transmit in ferrets, whereas PA I100V, K321N, V330I, and T639A increase the ability of GZ828 to transmit in ferrets (Meng et al., 2022). In addition, PA I38T reduces transmission among ferrets compared to WT viruses, and this relative cost of adaptation is higher in A/H1N1pdm09 viruses than in A/H3N2 viruses, where PA I38 is located on the plane of interaction with PB2 (Lee et al., 2021). In ferret transmission experiments, all viruses were transmitted to direct contact and airborne animals, except for the E23K+I38T virus, which was unable to infect 100% of animals by airborne transmission, and the PA E23G/K substitution weakened critical Baloxavir drug-binding contacts. The E23G/K virus has the ability to be airborne between ferrets. Therefore, there is a potential for community transmission of the E23G/K virus, which would adversely affect the clinical use of Baloxavir (Jones et al., 2022). In ferrets, H1N1 viruses with HA K154Q and PA L295P mutations showed significantly higher viral titers in the upper respiratory tract six days after infection, suggesting that two single mutations, HA K154Q and PA L295P, delayed clearance of H1N1 influenza viruses in a ferret animal model, and that the two mutations have a synergistic effect that also increases viral replication *in vitro* in NHBE cells (Ducatez et al., 2012). In addition, PA E23G, PA K34R, PA I38M/T, and the previously unreported PA A36T are essentially located in the PA-PB2 binding plane (Zhu et al., 2012; Andreev et al., 2024). PA K362R and S364N also enhanced transmission in avian species (Xu et al., 2023).

Influenza virus infection leads to a rapid decline in the overall synthesis of host proteins in infected cells, a process known as host shutdown (Khaperskyy et al., 2014). This process allows the virus to evade the host’s innate immune recognition and shuts down host antigen processing to disrupt acquired immunity, thereby allowing it to escape host restriction and facilitating its reproduction and transmission (Li et al., 2016). Influenza viruses activate multiple pattern recognition receptors (PRRs) to activate the innate immune response (Kiecolt-Glaser et al., 1996). Activation of these PRRs leads to further activation of downstream signaling pathways that result in the production of interferons (IFNs) and pro-inflammatory cytokines, followed by the expression of interferon-stimulated genes (ISGs), recruitment of innate immune cells or activation of programmed cell death (Xia et al., 2015). In innate immunity, Toll-like receptors (TLRs), Retinoic acid-inducible gene I (RIG-I), Nucleotide oligomerization domain (NOD)-like receptors (NLRs), Leucine-Rich Repeat signal pathway(LRR), pyridine domain-containing protein 3 (NLRP3), and Z-DNA binding protein 1 (ZBP1) play important roles in sensing and restricting influenza viral infection (Liu et al., 2007; Opitz et al., 2007; Allen et al., 2009; Goff et al., 2015; Kuriakose et al., 2016). Nuclear factor-kappa B (NF-κB) plays a crucial role in inflammation and immune response (Kumar et al., 2008; Ma et al., 2020). PA inhibits NF-κB transcription induced by poly (I: C) stimulation and also inhibits the expression of related genes regulated by this pathway (TNF-α, Nos2, IL-6). However, PA transcriptional repression of poly(I: C)-induced NF-κB was not characterized by an effect on NF-κB p65 activity. The transcriptional repression of NF-κB by PA was not linearly related to the expression of PA. Moreover, PA does not inhibit the phosphorylation process of the NF-κB pathway (Cui et al., 2017). IAV is recognized by RIG-I, which activates the type I interferon response and induces an antiviral effect (Wang et al., 2017). The PA protein inhibits Sendai virus-induced production of IFN-β and interferon-stimulated genes via interferon regulatory factor 3 (IRF3) (Bedsaul et al., 2016).PA is inhibited by its N-terminal endonuclease activity interacting with IRF3 to inhibit the IFN-β signaling pathway. In addition, PA inhibits melanoma differentiation-associated gene 5(MDA5), mitochondria antiviral signaling protein, and IRF3 overexpression (Benitez et al., 2015; Yi et al., 2017; Wu et al., 2024). The PA protein derived from the H5 subtype potently inhibits host antiviral defense by interacting with and degrading Janus kinase 1 (JAK1), a key protein for IFN signaling. In addition, the PA protein contained in 32T/550L degrades both avian and mammalian JAK1, whereas the PA protein contained in the 32M/550I residue degrades only avian JAK1. Furthermore, the 32T/550L site of the PA protein has the ideal polymerase activity for the growth of AIV in mammalian cells (Yang et al., 2023a).

PA-X shuts down host protein expression to inhibit the antiviral response (Hayashi et al., 2015). Its N-terminal 191 amino acids overlap with PA, while the first 197 aa of PA have endonuclease activity. Thus, PA-X can degrade host mRNA through endonuclease activity, preventing its translation and restricting host antiviral activity (Desmet et al., 2013). Gao et al. further demonstrated that the additional 20 amino acids at the C-terminus of the PA-X protein have a strong shutdown activity; six of these amino acids play a role in optimal host shutoff (Gao et al., 2015a). Gaucherand et al. discovered a unique mechanism by which PA-X tampers with RNA splicing to selectively disrupt host RNA (Gaucherand et al., 2019). The half-life of PA-X varies in different IAV strains, with most IAVs having short PA-X lifetimes. Sequences in the C-terminal domain of variable PA-X are primarily responsible for regulating the half-life of PA-X. PA-X from the 2009 pandemic H1N1 strain has a longer half-life than the other variants, and this PA-X heterodimer has higher host-shutdown activity, suggesting a role for protein turnover in the regulation of PA-X activity (Levene et al., 2021). Comparing the PA-X proteins of two related viruses, equine influenza(EIV) and canine influenza(CIV) H3N8, in which the EIV PA-X is about 50% more active in comparison with the CIV PA-X (Liu et al., 2020). Variability among strains in the activity of PA-X in shutting off the host and the residues in the N-terminal domain reflect the differences in PA-X activity. Interestingly, PA-X from avian viruses is more active than PA-X from human viruses, which may indicate that PA-X plays a role in optimal growth in a particular host (Levene et al., 2021). Hu et al. demonstrated that ablation of PA-X expression increases the virulence and replication of the H5N1 virus in mice and avian species and alters host innate immune and cell death responses. PA-X is a negative pathogenicity regulator that reduces pathogenicity by inhibiting viral replication and host innate immune responses, and expression of PA-X suppresses polymerase activity and viral replication *in vitro* and *in vivo* (Hu et al., 2016). The H1N1 PA-X expression deficiency can increase virus replication and apoptosis in A549 cells, as well as pathogenicity and host inflammatory response in mice (Gao et al., 2015b). Gao et al. found the absence of PA-X in the H9N2 virus reduced replication in human A549 cells. In addition, the absence of PA-X in the H9N2 virus attenuated the pathogenicity of the virus in mice (Gao et al., 2015c). Thus, unlike the previously reported H1N1 and H5N1 viruses, the PA-X protein in H9N2 viruses is a virulence-promoting factor that promotes viral pathogenicity, and the virulence-promoting or antiviral effects of PA-X in influenza viruses are strain-dependent. Competitive transmission studies in ferrets showed a replication advantage of 195K over 195R in H1N1/2009 viruses, indicating that the PA-X R195K mutation enhances transmission in ferrets. In contrast, PA-X 195K had no effect on the virulence of AIV H9N2 in chickens, suggesting that the effect of the substitution is mammalian-specific (Sun et al., 2020).

PA-X also plays an important role in the underlying regulatory mechanisms of the host’s innate immune response (Gaucherand et al., 2019). Previous studies have shown that PA-X reduces the pathogenicity of H5N1 Highly Pathogenic Avian Influenza Virus (HPAVI) by inhibiting viral replication and host response (Hu et al., 2015). PA-X inhibits poly(I:C)-induced NF-κB transcription, thereby reducing the expression of inflammatory factors related to NF-κB (Hu et al., 2020b). PA-X is demonstrated to suppress early accumulation of type I IFN mRNA, dependent on the activity of Mitochondrial Antiviral signaling (Rigby et al., 2019). Complete shutdown of host gene expression by PA-X may help the virus avoid immune recognition and appropriate antigen processing, thereby inducing acquired immunity. In view of such a strong influence of PA-X in the immune response, further analysis of the role of PA-X in immune regulation is required to unravel the mechanism of viral immune evasion for the development of effective influenza vaccines. The immense importance of dendritic cells(DCs) as a link between innate and acquired immune responses is clear because they are professional antigen-presenting cells (Helft et al., 2012). Inhibition of the PA-X protein of H9N2-infected chBM-DCs can inhibit the proliferation of T lymphocytes and subsequent downstream adaptive immune responses (Qin et al., 2023). The PA-X protein increases the virulence of the H1N1 virus through its ability to avoid the nasal mucosal DC immune response. The PA-X protein can inhibit the secretion of pro-inflammatory factors such as IL-6, TNF-α, and IL-1β while promoting the secretion of the anti-inflammatory factor IL-10 (Qin et al., 2022). It is well known that innate immunity is the first line of defense against viral infections; however, excessive innate immunity causes the secretion of large amounts of pro-inflammatory cytokines, leading to tissue damage in the host (Suzuki et al., 2009; Cilloniz et al., 2010; Hu et al., 2020b; Mulder et al., 2022). More interestingly, PA-X from H5N1 subtype AIV is able to reduce cytokine secretion by blocking the NF-κB signaling pathway and thus enables immune modulation, decreasing the virulence of influenza viruses (Hu et al., 2020b).

In a word, PA-X is an important element in innate immunity and plays a host-selective role against various influenza virus strains, which is a means of regulating innate immunity. In H1N1 and H9N2 influenza viruses, PA-X reduced DC infection and led to their phenotypic suppression; it functions as a pro-viral factor. However, in the case of H5N1 influenza virus infection, the function of PA-X was the opposite. Therefore, such a detailed mechanism of mucosal immunity mediated by PA-X needs a deep investigation.

## 4 Host factors interacting with influenza virus PA protein

PA is a major contributor to the propagation and growth of the influenza A virus (IAV) and is involved in several steps of the viral life cycle (Shapira et al., 2009; Yu et al., 2024). Investigating the host factors that interact with PA could assist us in comprehending the pathogenesis of influenza viruses more thoroughly. Currently, many studies commonly use immunoprecipitation-liquid chromatography-tandem mass spectrometry to identify hundreds of host proteins that may interact with IAV PA protein (Bradel-Tretheway et al., 2011; Yao et al., 2024). However, detailed mechanistic studies are scarce of the hundreds of host proteins interacting with PA analyzed by proteomics. Until now several dozen host factors interacting with PA proteins have been detailly documented. The PA protein influences the influenza virus life cycle in terms of nuclear import, replication, transcription, and effects on polymerase activity.

At first, several host factors interacting PA affect the nuclear importation processes of influenza viral proteins. Among these, Eukaryotic Translation Elongation Factor 1 Delta(eEF1D) was shown to be a very strong inhibitor of IAV replication, since its knockdown induced a significant increase in viral production. Moreover, eEF1D was found to interact in an RNA-dependent manner with RNP subunits PB2, PB1, PA, and other proteins. Further investigations demonstrated that eEF1D was able to control the nuclear infiltration of viral NP and PA-PB1 homodimers, which in turn controlled vRNP formation, polymerase activity, and the production of viral RNA (Gao et al., 2020). In order to investigate its interaction with host factors, a yeast two-hybrid screen was carried out with a cDNA library of HeLa cells. Results showed that PA interacts with HCLS1 associated protein X-1 (HAX1) through its nuclear localization signaling domain *in vivo*. The interaction with HAX1 prevents the nuclear transport of PA, thus inhibiting viral replication (Suzuki et al., 1997). Hsu et al. previously reported that HAX1 is an anti-apoptotic protein. HAX1 knockdown enhanced PA nuclear accumulation, while re-expression of HAX1 canceled this effect, which implies that HAX1 might block PA nuclear transport. Therefore, further study found that HAX1 inhibited by re-expression resulted in a significant increase in viral yield and polymerase activity. Therefore, the results indicated that HAX1 might restrict the replication of influenza A virus (Hsu et al., 2013). It was found that the interaction between IAV PA and Interferon regulatory factor 3 (IRF3) impeded the activation of IRF3 and thus blocked the IFN-β response pathway. The study showed a different tactic by which IAV limits the host IFN-β response pathway- the activity is mediated via the endonuclease domain of the PA-N terminus (Yi et al., 2017). Secondly, several host factors interacting with PA are important for the replication activity of viral polymerase during the influenza virus life cycle. CHD6 is a member of the CHD family of chromatin remodeling proteins, in which it has been found interacting with the viral polymerase complexes; hence, it is suggested that CHD6 acts as an influenza virus replication negative regulator (Alfonso et al., 2013). It is believed that the macrochromosome maintenance (MCM) complex, a DNA replication helicase enzyme, is a host factor that controls viral genome replication. Kawaguchi et al. found that the PA subunit of the influenza virus polymerase is a key factor in the transition from head-start to extension of the vRNA template, a process mediated by the host factor MCM Complex (Kawaguchi and Nagata, 2007). PKM2 interacts with the C-terminus of the PA subunit and enhances the replicative activity of influenza virus polymerase (Miyake et al., 2017). The ubiquitin A-52 residue ribosomal protein fusion product 1(UBA52) protein was screened through gene ontology and pathway enrichment analyses. UBA52 is a fusion protein that is a conserved host factor consisting of ubiquitin at the N-terminus and ribosomal protein L40 at the C-terminus. It can interact with PA, PA-N155, and PA-N182 proteins to promote viral replication. Knockout of UBA52 significantly reduces the titer of H5N1 IAV in chicken cells and reduces the production of pro-inflammatory cytokines (Wang et al., 2018). SNX2 reduces viral activity by affecting the replication of influenza virus polymerase (Koçmar et al., 2022). Thirdly, in addition to effects on replication, a number of host factors have an effect on the transcription activity of viral polymerase. The replication efficiency of highly pathogenic H5N1 avian influenza viruses in poultry is notably high, while in humans, it is comparatively low due to the distinctions between species in acidic nuclear phosphoprotein 32 (ANP32). The low polymerase activity of the PA protein drives the emergence of the H7N9 PB2 E627K (Liang et al., 2019; Arragain et al., 2024; Na et al., 2024). Carrique et al. analyzed the structure of human and chicken ANP32A complexes by cryoelectron microscopy and found that ANP32 is an important host protein for influenza virus polymerase, which binds to PA proteins and regulates viral gene transcription, enhancing influenza virus replication *in vivo* (Carrique et al., 2020). Histone Deacetylase 6(HDAC6), one critical site for PA stabilization, interacts with deacetylated PA and promotes its degradation via the proteasome pathway. Chen et al. demonstrated that HDAC6 restricts infection by IAV due to impacting the stability of the PA protein. HDAC6 overexpression suppresses the transcription of the IAV RNA polymerase. HDAC6 has a negative impact on the activity of RNA polymerase in IAV via deacetylation of PA, thereby reducing IAV RNA transcription and replication (Chen et al., 2019). SAM and HD domain containing deoxynucleoside triphosphate triphosphohydrolase 1 (SAMHD1) can restrict intracellular replication of H5N1, and the H5N1 PA protein can down-regulate SAMHD1 expression by affecting the activity of the SAMHD1 transcriptional promoter. They also found that the ability of SAMHD1 to restrict H5N1 is associated with the phosphorylation of 592-tyrosine (Zhao et al., 2024). Finally, some other host factors interacting with PA are involved in affecting polymerase activity to regulate influenza virus replication. Feng et al. identified a novel host factor, the aryl hydrocarbon receptor nuclear translocator (ARNT), it interacts with PA to reduce influenza virus polymerase activity and inhibit H5N1 virus replication (Feng et al., 2022). DnaJA1 interacts with RNA polymerase PA-PB1 to enhance viral replication (Cao et al., 2014). Additionally, Yang et al. found that advanced glycation end-product receptor 3(Galectin-3) could augment H5N1 virus-induced lung inflammation by activating NLRP3 inflammatory vesicles, which then had an effect on viral pathogenesis. Moreover, when expressed at higher levels, Galectin-1 and Galectin-3 act as either positive or negative regulators of innate defenses during inflammation (Yang et al., 2023b). Galectin-3, consisting of N-terminal ubiquitin and C-terminal ribosomal protein L40, binds to the PA subunit of the RdRp complex in infected lung epithelial cells, stimulating viral RNA synthesis (Obermann et al., 2017). Harte et al. further noted that CLE/C14orf166 protein (hCLE) is a 36 kDa protein encoded by the human gene. It was established that hCLE interacts with PA subunits in two sections of the PA protein sequence (493-512 and 557-574). The increase in hCLE protein levels throughout infection is dependent on polymerase activity, which promotes viral replication (Pérez-González et al., 2006). Gao et al. identified nucleolin (NCL) as a novel host protein that interacts with PA. Removal of endogenous NCL by siRNA targeting in mammalian cells during H5N1 virus infection resulted in a significant increase in viral polymerase activity. On the contrary, overexpression of NCL significantly reduced the replication of IAV, making NCL a novel potential antiviral factor during H5N1 infection. Further studies on the antiviral mechanism of NCL may accelerate the development of novel anti-influenza drugs (Gao et al., 2018). In addition, a wide array of host factors interacting with PA were identified (**Table 1**). Lastly, studying the interaction between host factors and PA protein will broaden our knowledge about the virus life cycle and provide novel ideas about the design of anti-influenza drugs. Understanding how and why influenza viruses damage cellular proteins and pathways will help us comprehend the action of host factors. The rapid evolution of influenza virus has caused the production of novel mutation sites for drug resistance. With host factors targeted drugs as the starting point, it is anticipated that more durable antiviral drugs will be created.

**Table 1:** Host factors interaction with influenza virus PA protein.

Host factors	Mechanism of action	Refs
ANP32A	Affect viral replication and transcription to enhance viral transcriptional activity	(Carrique et al., 2020)
ARNT	ARNT interacts strongly with the PA-C domain to inhibit viral replication	(Feng et al., 2022)
C14orf166	Promoting transcription of the viral genome	(Pérez-González et al., 2006)
COPI	Promoting viral replication	(Wang et al., 2016)
CHD6	A negative regulator of viral replication	(Alfonso et al., 2013)
DnaJA1	Interacting with RNA polymerase PA-PB1 to enhance viral replication	(Cao et al., 2014)
eEF1D	Inhibiting PA nuclear import to restrict viral replication	(Gao et al., 2020)
Galectin-3	Promoting viral RNA synthesis	(Obermann et al., 2017)
hCLE/CGI-99	Promoting replication of the influenza virus	(Pérez-González et al., 2006)
HAX1	Blocks nuclear transport of PA to prevent viral replication	(Hsu et al., 2013)
HDAC6	Negatively affects viral RNA polymerase activity by deacetylating PA	(Chen et al., 2019)
IRF3	Interacting with PA-N to inhibit viral replication	(Yi et al., 2017)
MCM complex	Initiate viral genome replication and stabilize RNA polymerase extension complexes	(Kawaguchi et al., 2011)
NCL	In the nucleus to inhibit viral replication	(Gao et al., 2018)
PKM2	Interacting with the C-terminus of the PA subunit to favor viral replication	(Miyake et al., 2017)
SAMHD1	Limit viral replication	(Zhao et al., 2024)
SNX2	Negatively affects avian influenza virus replication	(Koçmar et al., 2022)
UBA52	Interacting with PA to promote viral replication.	(Wang et al., 2018)

## 5 Application and research of antiviral drugs targeting PA and PA-X proteins

Influenza viruses cause significant economic losses to humans and livestock (Alkie et al., 2025). Vaccines and antiviral drugs are the two most effective strategies to prevent and control the pandemic of influenza viruses (Chiba et al., 2024). The approved anti-influenza viruses drugs are mainly categorized into inhibitors targeting the M2 ion channel, such as amantadine; inhibitors targeting neuraminidase, such as Oseltamivir; and inhibitors targeting the viral RNA polymerase, especially the N- terminal endonuclease domain of PA protein such as Baloxavir marboxil(BXM) and Favipiravir (Hay et al., 1985; Furuta et al., 2002; Rameix-Welti et al., 2006; Hayden et al., 2018). Since influenza viruses evolve rapidly, drug-resistant strains appear quickly once these drugs are used in clinics, resulting in less or no effectiveness. Therefore, the development of novel anti-influenza drugs is urgently needed.

The first generation of antiviral agents against influenza viruses is the inhibitors targeting the M2 ion channel, which mainly includes amantadine and rimantadine. The action mode of amantadine and rimantadine is to block the M2 ion channel and prevent viral uncoating, inhibiting the influenza viral life cycle (Belshe et al., 1988; Jing et al., 2008). Along with the widespread use in clinics, the resistant mutation arises rapidly. The L26F, V27A and S31N substitutions confer the resistance of influenza A viruses to amantadine and rimantadine (Gu et al., 2013; Musharrafieh et al., 2020). Since the resistant strains to M2 blockers exist widespread, the CDC reported that amantadine and rimantadine were not recommended as anti-influenza drugs (Belshe et al., 1989). An urgent requirement exists for novel medications to combat influenza viruses.

Neuraminidase inhibitors are mostly widely used in clinics for anti-influenza therapy. In 1999, the FDA approved neuraminidase inhibitor- oseltamivir as an anti-influenza (Hayden et al., 1999). Additionally, the approved neuraminidase inhibitors combating influenza are composed of Zanamivir, Peramivir, and Laninamivir (Monto et al., 1999; Kohno et al., 2010). Oseltamivir has a minimum duration of use only within 24 hours of symptom onset (Ng et al., 2010). The efficacy of oseltamivir in treating severe patients has been equally disappointing. Zanamivir is administered by nasal injection rather than oseltamivir, so its use is limited, especially in infants, children, and severely ill patients (Bergstrom et al., 1999). Peramivir is a recently approved drug for intravenous administration and is generally only available in hospitals (Chen et al., 2020). Laninamivir was approved in 2010 in Japan for combating influenza (Nakano et al., 2013). Zanamivir can only be used in inhalation (Cass et al., 1999). The mechanism of action of neuraminidase inhibitors is to inhibit virus release from the host cells (Vavricka et al., 2013). Although it was initially expected that influenza viruses would unlikely develop resistance to neuraminidase inhibitors, the H1N1 H275Y gene mutation in the Northern Hemisphere in 2007-2008 made it fully resistant to oseltamivir (Nakauchi et al., 2011). The clinical study shows that influenza viruses can evolve resistance without affecting their fitness for transmission (Leung et al., 2024). Currently, the number of influenza viruses that are unsusceptible to one or two kinds of approved neuraminidase inhibitors has already been raised (Wang et al., 2024). As shown in **Table 2**, in addition to H275Y (H274Y, N2 NA numbering) substitution, E119V, I222R, S246N, R292K and N294S were also associated with the resistance to oseltamivir of influenza A viruses (Okomo-Adhiambo et al., 2010; Li et al., 2012; Gillman et al., 2013). E119D/G, H274Y, S246G/R, and R292K reduce the efficacy of the anti-influenza drug Peramivir (Baek et al., 2015). Laninamivir and Zanamivir both were used for inhalation. NA E119D/G mutation results in the resistance of influenza viruses to these two NA inhibitors. Except for NA E119D/G, H274Y and R292K substitutions make the influenza viruses resistant to Laninamivir, while E119I, R152K Q136K, I222L, and S246R reduce the antiviral activity of Zanamivir (Kode et al., 2019). Above all, we know that NA E119 V/D/G/I mutation confers the resistance of all four approved NA inhibitors, and H274Y or R292K substitution makes three of four approved NA inhibitors inefficiency, including Laninamivir, Oseltamivir and Peramivir. Therefore, we can speculate that NA E119, H274 and R292 are the main targets of NA inhibitors. Hence, prompt sequencing of the genome of influenza viruses in the clinic would be of great value for antiviral selection. If we choose two NA inhibitors combination used in the clinic, one of Laninamivir, Oseltamivir or Peramivir combined with Zanamivir would be better than the others (Kositpantawong et al., 2021). Viral RNA-dependent RNA polymerase (RdRp), responsible for viral RNA transcription and replication, plays a crucial role in the life cycle of influenza viruses. Therefore, the polymerase complex is an ideal target for the development of novel anti-influenza drugs. Insight into the structure of the polymerase complex, together with functional investigation of polymerase subunits, has shown that the rational design of the molecule compounds to inhibit influenza viruses is reasonable (Yuan et al., 2016). The PA subunit is equipped with a nucleic acid endonuclease at its N-terminal (PA-N). Because nucleic acid endonuclease activity is of great importance in the viral life cycle, making it an ideal target for antiviral drugs (Watanabe et al., 2017). The N-terminal region of PB1 interacting with PA is remarkably conserved, suggesting a compound that can block the connection between the two proteins may be able to inhibit most influenza viruses. The docking results suggest that the binding of benzotriazole derivatives to the PA binding pocket of PB1 is possible by the requirement of the Trp706, Gln408, and Lys643 residues, thus enabling the formation of novel inhibitors of the PA-PB1 interaction (Pagano et al., 2014).

**Table 2:** Antiviral drugs against influenza A virus.

Name	Mechanism	Stage	Range	Route	Resistance resistance	Refs
Amantadine	M2 inhibitor	Approved	IAV	Oral	L27F V27A A30T S31N	(Stampolaki et al., 2023)
Rimantadine	M2 inhibitor	Approved	IAV	Oral	L27F V27A A30T S31N	(Galabov et al., 2006)
Laninamivir	NA inhibitor	Approved	IAV IBV	Inhalation	E119D/G H274Y R292K	(Kiso et al., 2023)
Oseltamivir	NA inhibitor	Approved	IAV IBV	Oral	E119V I222R S246N H274Y R292K N294S	(Okomo-Adhiambo et al., 2010)
Peramivir	NA inhibitor	Approved	IAV IBV	Injection	E119D/G R152K D198E S246G/R H274Y R292K	(Abed et al., 2010)
Zanamivir	NA inhibitor	Approved	IAV IBV	Inhalation	E119D/G E119I Q136K R152K I222L S246R	(Euzen et al., 2024)
Baloxavir marboxil	PA inhibitor	Approved	IAV IBV	Oral	E23K A37T I38T I38F E119G R142K D198E E199G	(Abed et al., 2020)
Anthralin	PA inhibitor	Approved	IAV	Injection	Unknown	(Hu et al., 2020a)
Favipiravir	PA inhibitor	Approved	IAV IBV	Oral	K229R(PB1) P653L(PA)	(Goldhill et al., 2018)
Arbidol	HA inhibitor	Approved	IAV	Oral	HA receptor-binding site and fusion peptide region	(Kadam and Wilson, 2017)
AL-794	PA inhibitor	In clinical	IAV IBV	Oral	Unknown	(Yogaratnam et al., 2019)
EGCG	PA inhibitor	In clinical	IAV	Oral	Unknown	(Ling et al., 2012)
RO-7	PA inhibitor	In clinical	IAV	Oral	T20A I79L F105S E119D	(Jones et al., 2017)
pimodivir	PB2 inhibitor	In clinical	IAV	Oral	F404Y M431I H357N	(Gregor et al., 2021)
PA-49	PA-PB1 inhibitor	In clinical	IAV IBV	cellular drug delivery	Unknown	(Watanabe et al., 2017)
Nitazoxanide	HA inhibitor	In clinical	IAV IBV	Oral	Unknown	(Haffizulla et al., 2014)

In 2018, BXM was approved by the FDA. It inhibits the influenza A and influenza B viruses and is highly effective in preventing the endonuclease activity of the viral PA subunit (Li et al., 2024). BXM is taken by oral administration and possesses high and broad potency for anti-seasonal influenza viruses (pdmH1N1, H3N2 and influenza B virus), avian influenza viruses, including H5N1, H7N9 subtypes, influenza C viruses and influenza D viruses (Mishin et al., 2019). BXM also effectively treats resistant variants with reduced NA inhibitor susceptibility (Arvia et al., 2025). Unfortunately, the resistance appeared during the clinical trial of BXM, and the study demonstrates PA I38T/F substitution confers the resistant profile of the influenza viruses to BXM (Wang et al., 2025). Coupled with the widespread use of BXM, more resistant mutations were identified, including the E23K, A37T, E119G, R142K, D198E and E199G of the PA subunit (Hickerson et al., 2022). Above all, BXM is an effective anti-influenza drug, especially for avian and resistant strains. Another approved anti-influenza drug is Favipiravir, which exerts its antiviral effect mainly by inhibiting the PA subunit and PB1 subunit of the influenza virus ribonucleoprotein, thereby inhibiting mRNA synthesis. Interaction with the viral RNA polymerase inhibits viral genome replication and transcription. In addition, Favipiravir nucleoside triphosphate can bind to the viral RNA strand, inducing lethal mutations during influenza virus infection, and reducing the virus titer *in vitro*, which exerts an antiviral effect (Sangawa et al., 2013). Favipiravir is administered orally, and the PA P653L and PB1 K229R mutations reduce the influenza virus’s susceptibility to the drug (Goldhill et al., 2018). In addition to the M2, NA and PA targets, HA is another antiviral drug target. The HA-targeted approved antiviral arbidol inhibits the HA-mediated membrane fusion to treat influenza virus infection (Li et al., 2022). Mutations in the receptor-binding site and the fusion peptide region of HA reduce arbidol susceptibility (Leneva et al., 2009).

The above are currently approved commercial antiviral drugs, with several other antiviral drugs still in clinical trials. Influenza virus polymerase remains a hotspot in antiviral drug development. AL-794 is administered orally and targets the PA subunit of the IAV, IBV influenza virus polymerase. In subjects, oral administration of 150 mg provided better protection than 50 mg. AL-794 was well tolerated, and no viral resistance was detected (Yogaratnam et al., 2019). Epigallocatechin gallate (EGCG), a green tea-derived polyphenol, exerted anti-influenza A virus activity *in vitro* and *in vivo*. EGCG exerted antiviral activity by inhibiting the PA-N (Ling et al., 2012). In addition, another PA endonuclease inhibitor, RO-7, protected mice from the lethal challenge of IAV, IBV. RO-7 treatment significantly reduced viral titers in the lungs and reduced the extent and severity of lung damage (Jones et al., 2017). However, the emergence of the T20A, I38T, I79L, F105S and E119D resistance sites reduced the susceptibility of RO-7 to the influenza virus. The I38T mutation site similarly reduced the therapeutic efficacy of BXM (Jones et al., 2018). The orally bioavailable small molecule JNJ4796 targets the conserved stem epitope on influenza HA to neutralize influenza virus by inhibiting HA-mediated fusion. The compound mimics key interactions observed in antibody-HA co-crystal structures, inhibits pH-sensitive conformational changes of HA, neutralizes influenza virus *in vitro* and protects mice from lethal virus challenge (van Dongen et al., 2019). We have summarized the antiviral drugs that have been approved and are currently in the clinical stage, to provide a reference for the research and development of antiviral drugs (**Table 2**).

## 6 Conclusion

Recently, influenza viruses have swept the world, causing a significant impact on humans and poultry. Strains represented by the Influenza virus A (H1N1) pdm09 subtype pose a threat to human health, while the H5N1 strain, which is represented by the rapid spread among dairy bovine in the United States, has also been found to be transmitted from bovine to humans. The PA protein plays an important role in the influenza virus polymerase with endonuclease activity and multiple functional domains. The PA-X protein is a truncated form encoded by the PA protein and significantly affects host gene expression and viral pathogenicity. PA and PA-X play important roles in regulating viral replication, transcription, pathogenicity and host immune evasion, and these functions make PA and PA-X attractive targets for developing antiviral drugs. The effective drugs targeting PA proteins are Baloxavir marboxil and Favipiravir, but resistant influenza viruses have emerged with the appearance of drug-resistant mutation sites. In this process, the interaction of host factors with PA makes a significant impact on the virus life cycle; therefore, targeting host factors with antiviral drugs will most likely be the next target for intervention. It would provide insights into the molecular mechanisms underlying the pathogenesis of influenza infection due to complex interactions among PA with host factors. Due to the constantly evolving threat of influenza viruses, further investigation of virus properties remains important to decreasing the burden of influenza pandemics. This review discusses the structural characteristics, functional roles, immune regulation, host factors and the latest advancements in the development of antiviral therapies targeting the influenza virus PA and PA-X. It serves as a valuable reference for future efforts in controlling and preventing influenza outbreaks and for the development of novel antiviral drugs and vaccines.
